# Reduced False Positives and Improved Reporting of Loop-Mediated Isothermal Amplification using Quenched Fluorescent Primers

**DOI:** 10.1038/s41598-019-43817-z

**Published:** 2019-05-14

**Authors:** Patrick Hardinge, James A. H. Murray

**Affiliations:** Cardiff School of Biosciences, Biomedical Science Building, Museum Avenue, Cardiff, CF10 3AX UK

**Keywords:** DNA, Biochemical assays, DNA synthesis

## Abstract

Loop-mediated isothermal amplification (LAMP) is increasingly used in molecular diagnostics as an alternative to PCR based methods. There are numerous reported techniques to detect the LAMP amplification including turbidity, bioluminescence and intercalating fluorescent dyes. In this report we show that quenched fluorescent labels on various LAMP primers can be used to quantify and detect target DNA molecules down to single copy numbers. By selecting different fluorophores, this method can be simply multiplexed. Moreover this highly specific LAMP detection technique can reduce the incidence of false positives originating from mispriming events. Attribution of these events to particular primers will help inform and improve LAMP primer design.

## Introduction

In molecular diagnostics, isothermal nucleic acid amplification methods are an attractive alternative to benchmark polymerase chain reaction (PCR)^1^ strategies because of the low cost equipment required to initiate and maintain the reaction. This is of particular relevance to low resource settings and point-of-care applications. Numerous methods have been described^2–4^ for isothermal amplification of which loop-mediated amplification (LAMP)^5^ is the most widely reported. LAMP based assays have been used for numerous applications including the detection of pathogens such as malaria^6^ and salmonella^7^, viral RNA rapid detection for HIV with reverse transcriptase LAMP (RT-LAMP)^8^, GM crop contamination^9^ and in forensic science to specifically detect human DNA^10^.

**Figure 1:**
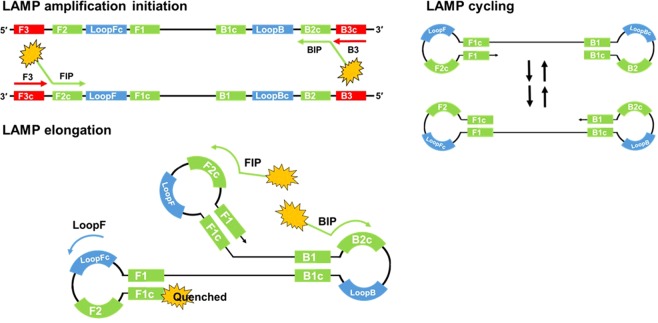
LAMP amplification initiation, cycling and elongation. Strand invasion of double stranded DNA with LAMP and displacement primers initiates the LAMP reaction. Positions of primer recognition sites are shown in red for displacement primers, green for LAMP primers and blue for Loop primers. LAMP initiation leads to the formation of dumbbell-like structures with cycling between the two forms and elongation into cauliflower-like structures with multiple loops. The fluorophore is shown unquenched attached to the FIP primer and quenched by proximity to guanine bases due to fluorescence resonance energy transfer (FRET).

The LAMP reaction is initiated by strand invasion of the DNA template by hairpin-forming LAMP primers which anneal and extend catalysed by a strand displacing DNA polymerase. These annealed LAMP primers are in turn displaced by displacement primers in the initiation of amplification, and lead to the formation of a dumbbell-like structure. This structure forms the basis of cycle amplification and elongation, with only LAMP primers, into cauliflower-like formations of single stranded DNA loops. The LAMP primers FIP and BIP (forward and backward inner primer), are designed to hybridise to the complementary and reverse complementary target sequences of F2/B2 and F1/B1 (Fig. 1). Displacement primers are referred to as F3 and B3. The addition to the reaction of Loop primers^11^ and STEM primers^12^ accelerates the DNA amplification by hybridising and extending from the hairpin loops or the region between loops respectively. Faster reaction times have been shown with the addition of swarm primers^13^. LAMP amplification is typically achieved at 60 to 65 degrees C for a time period dependent on the concentration of the template. Highly desirable characteristics of LAMP includes high sensitivity and specificity with rapid reaction times and there is also evidence that LAMP amplification will proceed in the presence of PCR inhibitors^9,14,15^ permitting less stringent DNA extraction procedures.

PCR has been developed to increase the specificity of the two primer amplification method. Real time PCR^16^ developments have included amplicon specific TaqMan probes^17^ which are quenched when unbound, but the fluorophore is unquenched by release from the probe because of the 5′ to 3′ exonuclease activity of Taq polymerase. Molecular beacons^18^ are self-quenching oligonucleotides with a template specific loop structure which when bound to the target sequence unquenches the fluorophore, unbound molecular beacons remain quenched. The increased specificity of these example methods, combats the false positive results that can affect indirect methods of amplification detection such as intercalating dyes and gel electrophoresis.

Although LAMP isothermal amplification with Loop acceleration utilises six primers targeting eight target DNA sequences, the incidence of false positive results has been reported^19^. LAMP assays may use indirect methods of amplification detection and these, whether real-time or end-point, rely on careful primer design, optimal reaction conditions and robustness testing to negate false positives. Such indirect detection strategies include the addition of intercalating dyes for fluorescence or colorimetric determination, turbidity^20^ from pyrophosphate precipitation, observations of LAMP ladder patterns with agarose gel electrophoresis^5^, quenched calcein^21^, hydroxy-naphthol blue^22^ as well as a number of fluorescent probe based techniques^15,23,24^. Real-time methods include bioluminescent reporting (BART)^25^ whereby the increase in inorganic pyrophosphate during nucleic acid amplification is converted to ATP which is converted into detectable light using a thermostable luciferase and luciferin substrate. The incorporation of intercalating dyes such as SYBR Green^26,27^ and the SYTO family^28,29^ generate increased fluorescence with increasing double-stranded DNA propagation. BART and SYTO9 real time LAMP amplification detection are used in this report. Other real time methods include turbidimetry^30^, colour change with pH sensitive dyes^31^ and amplifying with the nucleic acid specific dye berberine^32^.

To improve the specificity of LAMP, a number of solutions involving fluorescent labelled probes and primers have been proposed. Fluorescent probes used for quantitative PCR such as for example molecular beacons^33^, have been adapted to LAMP, however the displacement activity of the polymerase and lack of thermal cycling make such adaptations problematic. Fluorescently labelled oligonucleotide probes targeting the LAMP loop structures^34^ offer increased specificity when visualised after precipitation with polyethylenimine (PEI) which forms an insoluble fluorescent complex. Proximity to guanine nucleotides had been previously shown to quench certain fluorophores^35^ and forms the basis of a number of specific LAMP detection methods. Alternately binding quenching probe competitive LAMP (ABC-LAMP)^15^ is another oligonucleotide probe method for increased specificity, whereby the AB-Q probe with a 5′ fluorophore hybridises to the target loop and specially designed competitor DNA which has a number of guanines replaced with cytosines in proximity to the fluorophore. Real time LAMP amplification will therefore show a reduction in fluorescence in the presence of specific loop sequences. Fluorescent labelling of the loopF primers for fluorescence quenching during LAMP amplification by proximity to guanine^36,37^ enabled different target LAMP amplifications to be detected. Further multiplex detections were shown with melt LAMP (mLAMP)^38,39^ designed to use fluorescent labelled FIP primers for end point detection following real time turbidimetry of multiple targets.

Fluorescence resonance energy transfer (FRET) methods such as duplex LAMP^23^ utilise an acceptor intercalating dye and 5′ end FAM labelled BIP primer for one gene target sequence and no fluorophore for another. Increase or decrease in fluorescence is indicative of the gene present. A FRET LAMP assay^19^ utilises a combination of donor probe, acceptor probe and a hybridisation probe to detect white spot syndrome virus by fluorescence. Another probe method for increased specificity is the detection of amplification by release of quenching technique (DARQ)^40^ whereby a quencher linked to the 5′ end of a FIP primer quenches a hybridised fluorescent probe which is released during amplification. Assimilating probe^24^ employs a similar methodology. One step strand displacement LAMP amplification (LAMP-OSD)^41^ utilises a quenched reporter consisting of Reporter F and Reporter Q probes. Reporter F has an additional 11 nucleotide toehold sequence and the probe hybridises to a specific target loop of the amplification. The probe is unquenched and fluorescence can be detected. Graphene Oxide based FRET LAMP^42^ is another approach that uses a fluorescent probe, but with graphene oxide quenching the DNA fluorophore complex. Quenching of unincorporated amplification signal reporters (QUASR)^43^ offers an alternative approach for increased specificity using temperature to determine the presence of target. Labelling FIP and BIP primers with various fluorophores and dot-ELISA^44^ was used in a further specific multiplex LAMP assay and recently fluorescence of Loop primer upon self dequenching (FLOS LAMP)^45^ used modified thymine residues towards the 3′ end of primers with linked fluorophores so that during LAMP amplification the probes were unquenched.

In our work we have combined the 5′ fluorescent labelling of LAMP primers with quenching by LAMP amplification to provide a simple and specific method of DNA quantification and detection. The LAMP primers are ideally suited to this purpose because they are both essential and lead to the formation of complex looped structures. The attachment of a fluorophore does not appear to compromise LAMP target sensitivity and detection times remain rapid when compared to qPCR. We further show how the labelling of different primers, combined with non-specific detection of amplification with SYTO9 can be used to inform the primer optimisation process in designing LAMP assays. Furthermore we show that multiplex assays are possible with this method.

## Results

**Figure 2:**
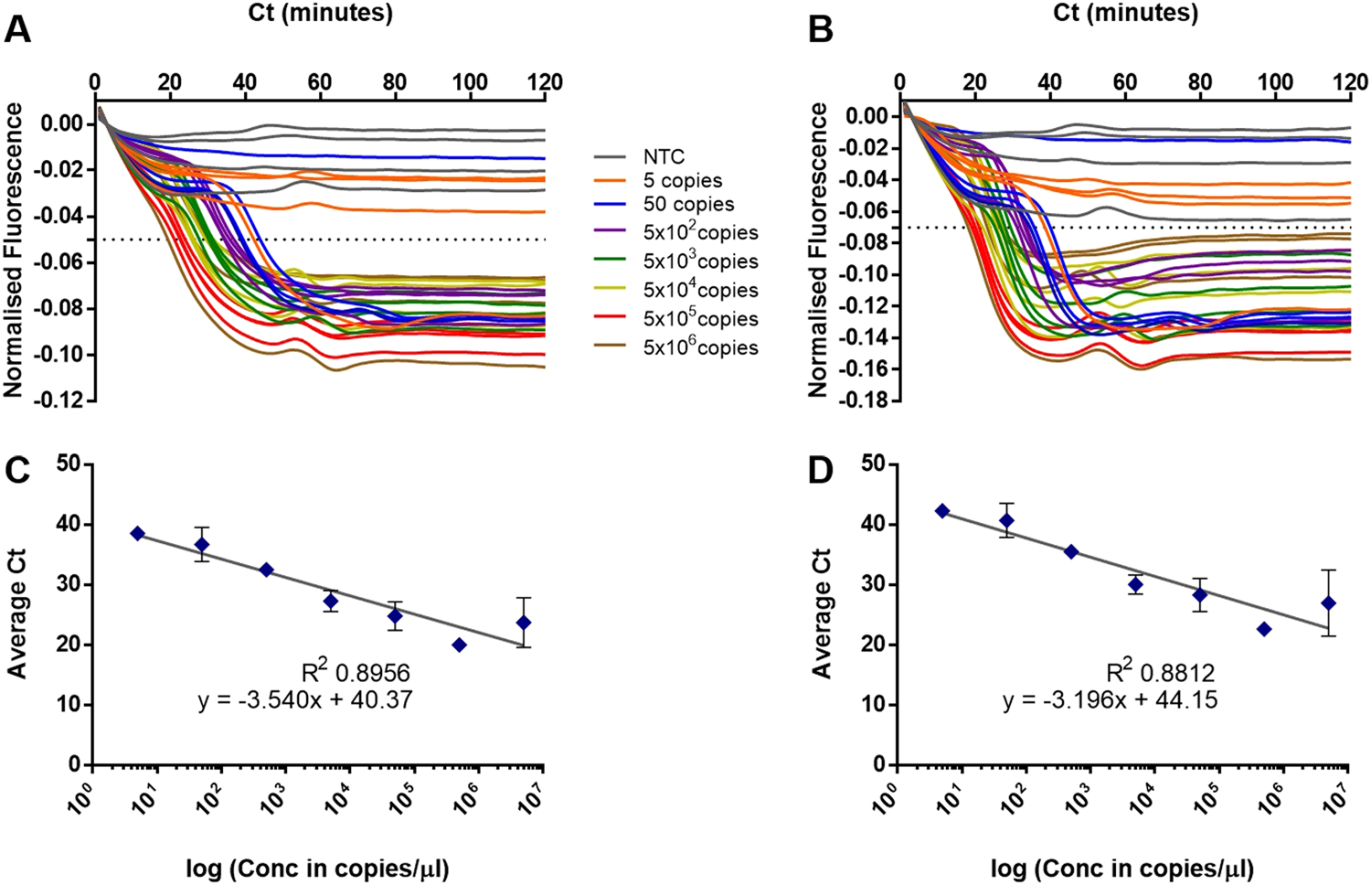
Quenching of fluorescence. LAMP amplification with 35S promoter primers substituted with a FIP primer labelled with JOE and LoopF primer labelled with FAM. Dilution series of a linear plasmid template and with additional no template control. Quenching of the FAM signal from the FAM-LoopF primer (**A**) and quenching of the JOE signal from the JOE-FIP primer (**B**). Four replicates for each dilution of template and for NTCs. Average Ct (cycle threshold set at −0.05 or −0.07) against logarithmic scale of the template dilutions for JOE-FIP (**C**) and FAM-LoopF assays (**D**). Three out of four samples were positive at 50 copies, one out of four samples were positive at 5 copies. The R2 values with the highest concentration of template removed were 0.9868 and 0.9795 for JOE-FIP and FAM-LoopF respectively.

The ‘cauliflower-like’ structures which are rapidly generated in LAMP amplification potentially bring into proximity the 5′ end of a Loop primer with the 5′ end of a LAMP primer (Fig. 1). We investigated using a FAM labelled LoopF and a JOE labelled FIP to explore whether fluorescence resonance energy transfer (FRET) could be observed between these two fluorophores by excitation of FAM transferring energy to JOE for increased fluorescence (Fig. 2). However we observed a decrease in fluorescence with time from JOE and upon further investigation it was clear that the fluorescence of both of these fluorophores was quenched during the LAMP amplification. Furthermore the time at which quenching occurred was proportional to the original concentration of the DNA template, suggesting it was linked to the increasing amount of LAMP amplicon.

The results in Fig. 2 show that the labeling of either the FIP primer or the LoopF primer produced similar results in terms of degree of signal quenching and the timing of the quenching. The non-template controls remained constant throughout the assay time of 120 minutes. All four replicates of samples with 500 copies or more of linear plasmid template were detected, but only 75 percent of the samples with 50 copies and 25 percent of those with 5 copies. However the positive results at these low concentrations of template were closely aligned with quantitative semi-logarithmic trend lines (Fig. 2C,D).

**Figure 3:**
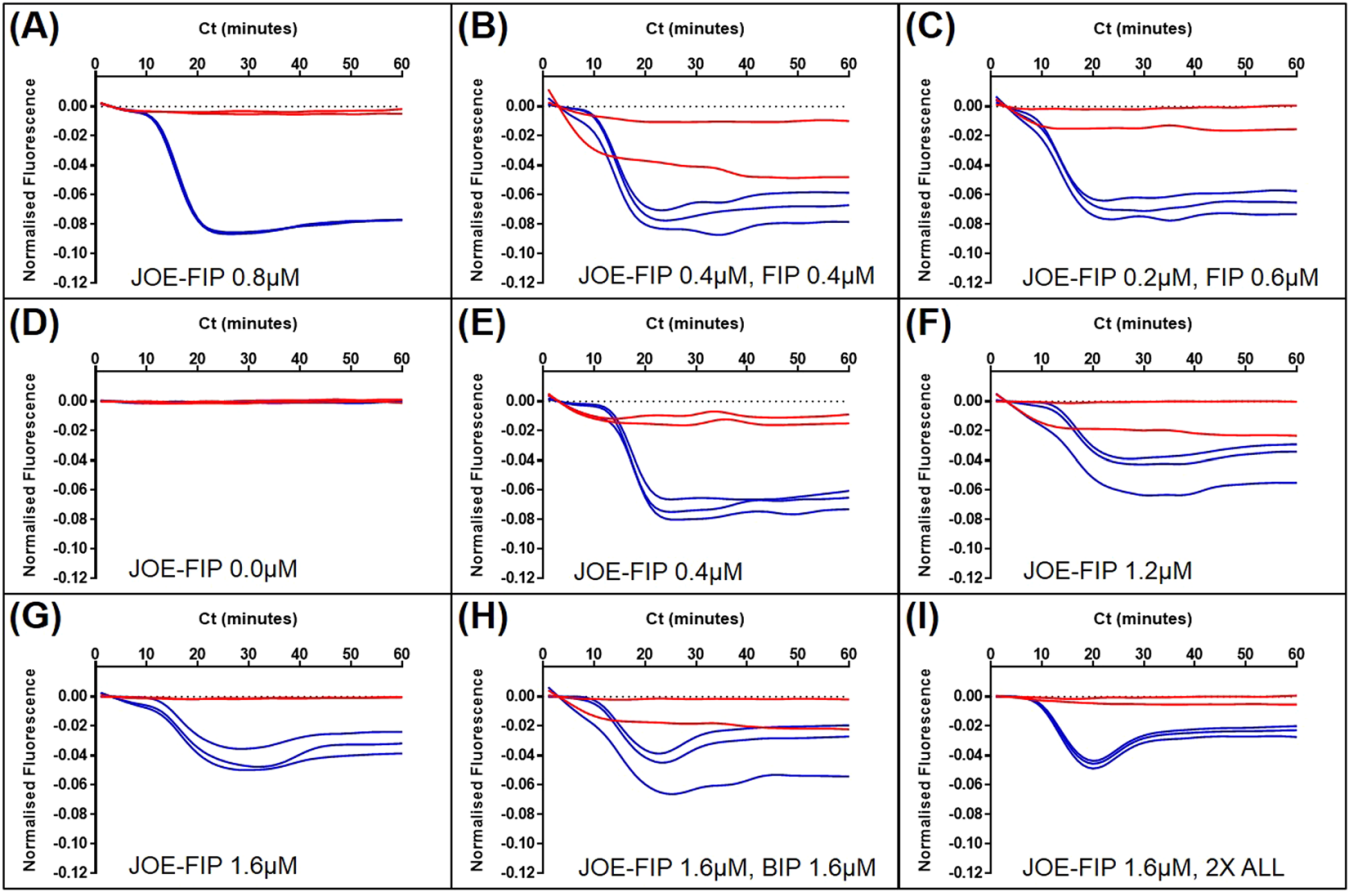
LAMP primer combinations with JOE labelled FIP. LAMP amplification and fluorescent detection of 50000 copies per 5 microlitres linear plasmid DNA using 35Sp primers; (**A**) 0.8 micromolar JOE-FIP with 1x other primers (0.8 micromolar BIP, 0.4 micromolar LoopF and LoopB, 0.2 micromolar F3 and B3), (**B**) 0.4 micromolar JOE-FIP, 0.4 micromolar FIP, 1x other primers, (**C**) 0.2 micromolar JOE-FIP, 0.6 micromolar FIP, 1x other primers, (**D**) no fluorescently labelled primers, 1x primers, (**E**) 0.4 micromolar JOE-FIP, 1x other primers, (**F**) 1.2 micromolar JOE-FIP, 1x other primers, (**G**) 1.6 micromolar JOE-FIP, 1x other primers, (**H**) 1.6 micromolar JOE-FIP, 1.6 micromolar BIP, 1x other primers, (**I**) 1.6 micromolar JOE-FIP, 2x other primers. Red lines: no template control; blue lines: samples with 50000 copies of target.

The concentration of primers and the ratio of primer combinations which contain the fluorescently labelled FIP were then optimised and shown in Fig. 3. Best results were obtained with 0.4 to 0.8 micromolar JOE-FIP (Fig. 3A,E).

The clearest separation between the detection of amplified template and the negative template controls (NTC) coupled with low variation between the replicates was observed with 0.8 micromolar JOE-FIP and the standard concentration of the other primers (Fig. 3A). The concentration of 0.4 micromolar JOE-FIP with standard concentration of the other primers (Fig. 3E) was better separated with less replicate variation than 0.4 micromolar JOE-FIP with 0.4 micromolar FIP (Fig. 3B). However 0.2 micromolar JOE-FIP with 0.6 micromolar FIP (Fig. 3C) was also better than 0.4 micromolar JOE-FIP and FIP. The higher concentration of JOE-FIP of 1.6 micromolar (Fig. 3G,H,J) all showed reduced initial quenching when compared to the lower concentration of JOE-FIP with a further reduction in quenching towards the end of the assay.

**Figure 4:**
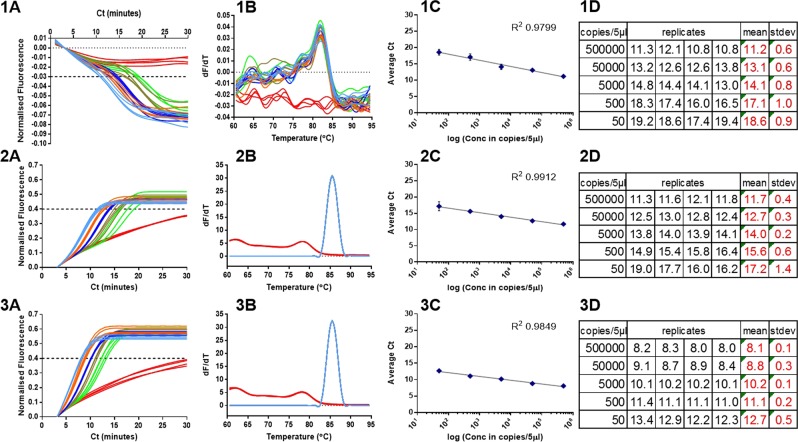
Detection with JOE-FIP and SYTO9. LAMP amplification with 35S promoter primers of a linearised plasmid template (1) quenched fluorescence in presence of JOE-FIP primer with SYTO9 using yellow channel detection of JOE, (2) fluorescence of same samples with green channel detection of SYTO9, (3) fluorescence with unmodified primers with SYTO9 only (green channel). (**A**) Normalised fluorescence truncated from 60 minute assay, (**B**) melt curve analysis, (**C**) average Ct plotted against template concentration on a log scale and (**D**) summary of replicate Ct values, average Ct and standard deviation (SD) for each dilution. NTCs (red lines) were negative. Light blue lines: 500000 copies; orange: 50000 copies; dark blue: 5000 copies; brown: 500 copies; green: 50 copies.

Using the optimised ratio and concentration of primers in Fig. 3A the quenching LAMP method was compared to LAMP detection with the intercalating dye SYTO9. The SYTO9 was used in the presence or absence of JOE-FIP primer to gauge the impact of the labelled primer on the assay kinetics. In Fig. 4,1, the assay with the JOE-FIP primer in the presence of SYTO9 was acquired in the yellow channel to detect the fluorescence signal from JOE, and the assay with 50000 template copies had an average Ct of 11.2 (SD 0.6) and for 50 copies an average Ct of 18.6 (SD 0.9), at a defined cycle threshold of −0.03. In Fig. 4,2, the JOE-FIP primer with SYTO9 assay was acquired via the green channel to detect the fluorescent signal from SYTO9. The 50000 template copies assay had an average Ct of 11.7 (SD 0.4) and for 50 copies this figure was 17.2 (SD 1.4) at a cycle threshold of 0.4. The replicate assay with unlabelled FIP and SYTO dye had more rapid Ct values of 8.1 (SD 0.1) for the 50000 copies and 12.7 (SD 0.5) for the 50 copies at the cycle threshold of 0.4 (Fig. 4,3).

The linearity for the dilution range to a semi-log model with each method was high (R2 0.9799 to 0.9912), however it is apparent that the variation between replicates increases with decreasing concentration as previously observed^46^. The variability between replicates at each dilution for the SYTO9 detection method was lower than that of the quenched fluorophore technique and increased variation and slower detection times were shown with SYTO9 detection using the primer combination that included the fluorophore labelled FIP. Melt curve analysis showed almost identical profiles for the two assays with SYTO9 detection and the melt curve using quenched fluorophore detection clearly separates the positive from the NTC replicates.

**Figure 5:**
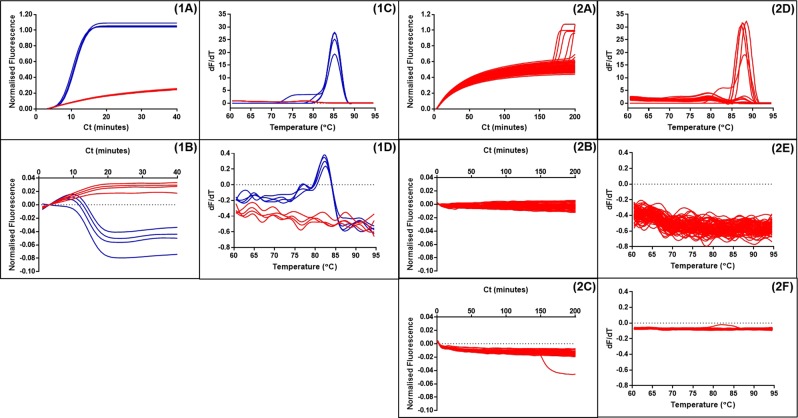
Detection with SYTO9 and JOE-FIP positive and negative samples. (1) LAMP amplification of 500000 copies linear plasmid template and NTCs with detection by both (**1A**) and JOE-FIP quenched fluorescence (**1B**) in separate tubes. Four replicates of template in blue and NTCs in red. Melt curve analysis (**1C,D**) showing amplification of template only in the 90 minute assay. (2) Amplification with LAMP primers of 72 replicate NTCs for 200 minutes. (**2A**) SYTO9 detection of amplification between 150 and 200 minutes showing 5 of 72 giving a positive signal. (**2B**) JOE-FIP detection of the same samples using the yellow channel (excitation at 530 +/− 5 nm with emission at 555 nm +/− 5 nm). (**2C**) FAM-LoopF detection using the green channel (excitation at 470 nm +/− 10 nm with emission at 510 nm +/− 5 nm). (**2D–F**) Melt curve analyses.

The quenched fluorophore method using labelled LoopF or FIP primers could provide an enhanced specificity when compared to detection methods that rely solely on increases in double stranded DNA or pyrophosphate production, because only the products of LAMP amplification will be detected and not ‘false positives’ from non-specific primer interactions. To investigate this possibility LAMP primers with minimal post-synthesis purification were subjected to extended assay times of 200 minutes to encourage amplification from non-specific primer interactions (Fig. 5,2A–C).

Using SYTO9 for detection, five of the seventy two NTC replicates gave a positive signal between 150 and 200 minutes (Fig. 5,2A). The melt curve analysis (Fig. 5,2D) showed a higher Tm than for the true LAMP products formed from template with the same primers (Fig. 5,1C) with SYTO9 detection. The LAMP assay with JOE labelled FIP supplemented for the basic purification FIP (Fig. 5,2B) showed no positive melt curve results from the same seventy two NTCs over the 200 minute assay time, when compared with the corresponding LAMP assay with template and NTCs (Fig. 5,1B). The supplementing of LoopF with FAM labelled LoopF showed one of the seventy two NTCs as a positive (Fig. 5,2C). We conclude that combining SYTO9 detection with quenched fluorophore detection provides two methods of detection in one tube at the same time increasing overall specificity.

We expanded this concept to see if we could observe simultaneous non-specific detection with SYTO9 and specific detection with the quenched fluorophore attached to the FIP LAMP primer. Using JOE to label the FIP primer, fluorescence acquisition can be achieved with excitation and emission of SYTO9 at 470 and 510 nanometers separated from JOE excitation and emission at 520 and 555 nanometers. Assay times were extended to 180 minutes to facilitate non-specific primer interactions.

**Figure 6:**
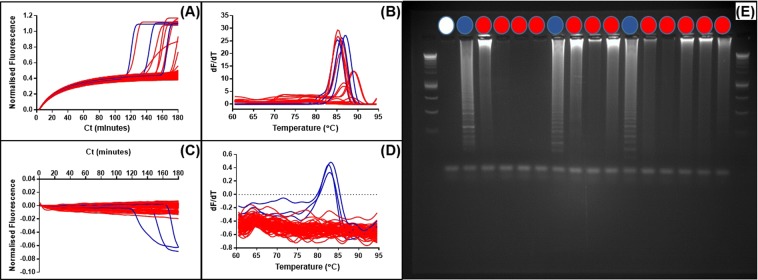
Non-specific primer interactions detected with SYTO9 and JOE-FIP. Panel (A) shows the normalised fluorescence from SYTO9 detection and (**C**) shows the consecutive JOE-FIP fluorescence for NTCs. Corresponding melt curve analysis shown in panels (B,D). Negative results and SYTO9 detected positives are highlighted in red, JOE-FIP detected positives are highlighted in blue. One negative and all positives were examined on an agarose gel (**E**) for amplicons and the JOE-FIP detected positives showed a ladder pattern associated with LAMP amplification.

The detection of the fluorescence from the JOE labelled FIP primer in the LAMP amplification showed three positive results from the seventy two NTCs. These positives were detected after 100 minutes (Fig. 6C). Detection of the same LAMP amplification from the seventy two NTCs with SYTO9 (Fig. 6A) showed a higher number of positives with the three positives detected by the quenched fluorophore method also positive with SYTO9 detection (highlighted in blue). Melt curve analysis showed a spread of melt temperatures for these amplicons between 85 and 90 degrees C. However agarose gel electrophoresis (Fig. S1) showed LAMP ladder patterns for the three positives detected using the JOE labelled FIP primer in contrast to the other putative positives detected only using SYTO9. The detection of the three ‘false positives’ which produced a ladder pattern similar to that seen from LAMP amplicons suggest that the JOE labelled FIP primer was one of the primers involved in the non-specific primer interaction.

**Figure 7:**
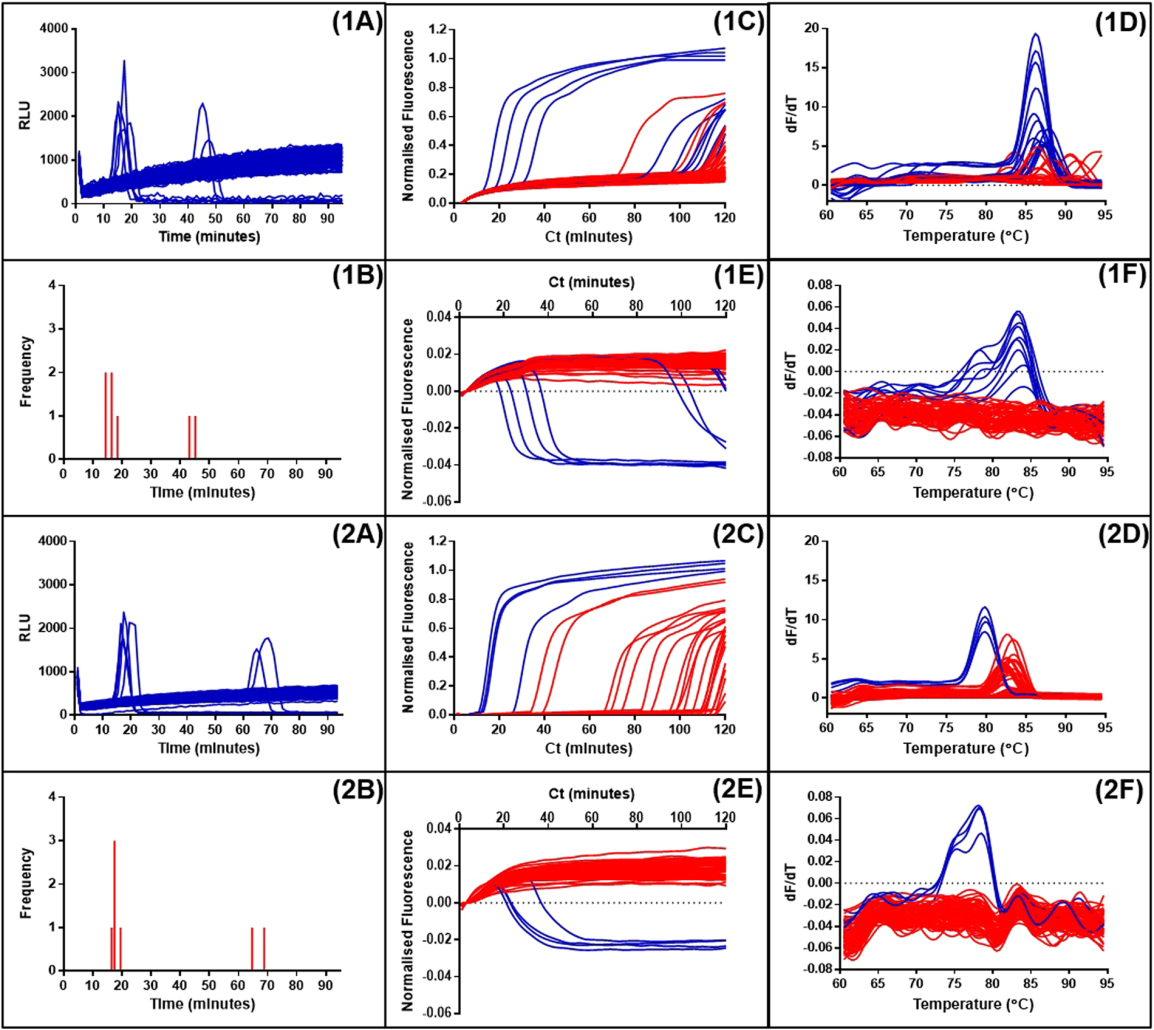
Detection of LAMP amplification with SYTO9 and JOE-FIP reporting compared to LAMP-BART results. Panels (1A) to (1F) represent amplification with the 35S promoter primers of a low concentration of denatured maize genomic DNA (0.1 percent maize GM event Bt11). Panels (2A) to (2F) represent amplification of the same sample with the NOS terminator primers. Panels (1A and 2A) show LAMP amplification with BART detection with corresponding frequency distribution of positive results (**1B** and **2B**). Panels (1C and 2C) show detection using SYTO9 and (**1E** and **2E**) show the simultaneous results from JOE-FIP detection. The melt curve analysis for these results are shown in panels (1D,1F,2D,2F).

At higher concentrations of template the importance of non-specific primer interactions is likely to be less significant due to the domination of ‘true’ LAMP amplification. However at very low concentrations of template, and for example the template dilutions used in digital quantification^47,48^, the prevalence of ‘false positives’ will be detrimental to accurate quantifications. We therefore investigated dual detection of dilute samples of denatured maize genomic DNA with a low proportion of transgenic targets to compare sensitivity to bioluminescent detection of pyrophosphate production (BART) and to identify incidence of non-specific amplification (Fig. 7).

LAMP BART assays of a denatured maize genomic DNA sample showed five positive results between 10 and 20 minutes and a further two positive results after 40 minutes within the 90 minute assay period, with two different primer sets targeted to the 35S promoter and NOS terminator. The dual detection using 35Sp primers and JOE-FIP highlighted six positives before 90 minutes with the majority between 10 and 40 minutes. One of the positives was detected by SYTO9 only and is therefore likely to be a false positive. The sensitivity between the LAMP BART and the dual detection methods appeared to be similar. With the NOSt LAMP dual detection the quenched fluorescence detected four positive results all between 10 and 40 minutes. The SYTO9 method detected numerous other positives but the melt curve (Fig. 7,2D) indicated that these products have a higher melt temperature than the ones identified with the quenched fluorescence method.

Carrier DNA is often used in LAMP assays to improve results^9^, however the mechanism by which this happens is unclear. We therefore used the dual detection method to investigate whether the addition of carrier DNA increases the incidence of non-specific primer interactions, thereby only apparently increasing the amplification frequency.

**Figure 8:**
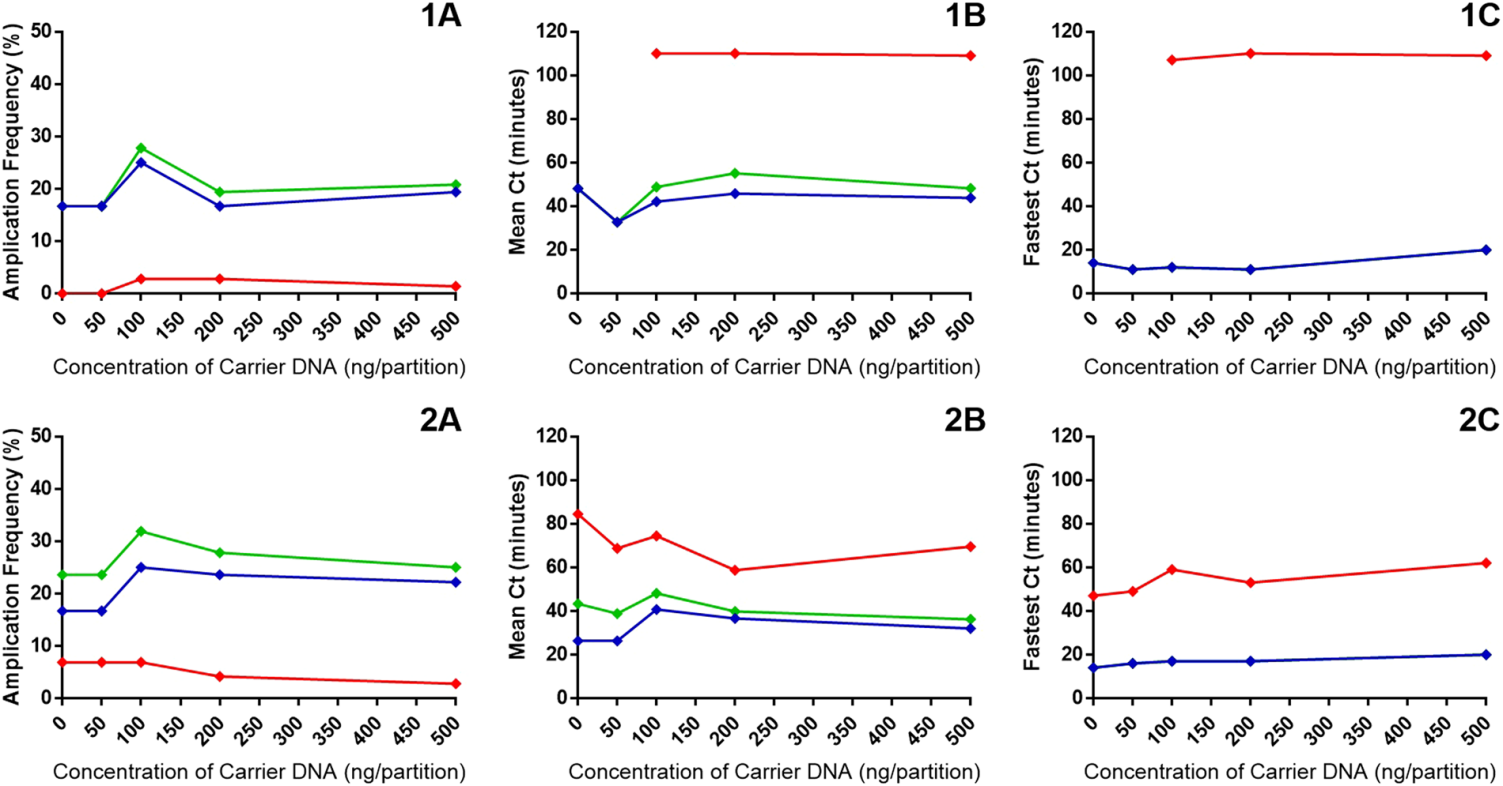
Carrier DNA in LAMP amplification with SYTO9 and JOE-FIP detection. Panels (1A to C) represent amplification with the 35S promoter primers of a low concentration of denatured maize genomic DNA (5.0 percent maize GM event Bt11). Panels (2A to C) represent amplification of the same sample with the NOS terminator primers. Panels (A) show the amplification frequency for increasing concentrations of carrier DNA. Cycle threshold set for all assays at −0.005. Panels (B) show the average Ct values and panels (C) shows the fastest Ct values with increasing concentrations of salmon sperm carrier DNA. Green indicates SYTO9 derived values, blue indicates JOE-FIP derived values and red indicates ‘false positive’ results derived from the difference between the SYTO9 and JOE-FIP values. In panels 1C and 2C the SYTO9 and JOE-FIP values are identical.

The amplification frequency of both LAMP primer interrogations of the same template showed the frequency increased using JOE-FIP quenched fluorescence detection from 18 percent without carrier DNA to approximately 25 percent with 100 nanograms per partition of salmon sperm carrier DNA (Fig. 8A). There was a general trend downwards of ‘false positives’ (in red) with increasing concentrations of carrier DNA indicating that non-specific primer interactions are not contributing to the improved amplification frequency seen with carrier DNA inclusion. For both primer sets with this template the optimum concentration of carrier DNA is 100 nanograms per partition. However, the average Ct values for both primer sets with JOE-FIP detection are faster without carrier or with 50 nanograms per partition of carrier DNA than the 100 nanogram level. The fastest Ct value at each concentration of carrier DNA with both LAMP primer sets remains fairly constant below the 20 minutes level. This result taken in conjunction with the average Ct values suggests that carrier DNA produces greater variability between replicates.

**Figure 9:**
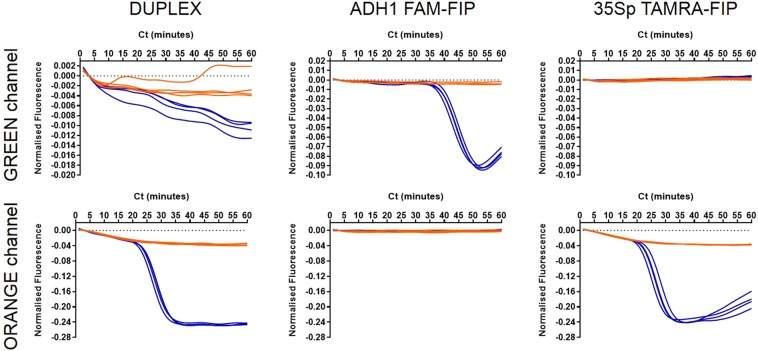
Duplexed LAMP reaction with ADH1 and 35Sp detection. LAMP amplification and detection of 20 copies of denatured Bt11 maize genomic DNA with ADH1 LAMP primers with FAM labelled FIP and 35Sp LAMP primers with TAMRA labelled FIP. Blue indicates template amplification and orange denotes the NTCs. The (DUPLEX) column shows the detection of two LAMP reactions in each tube. Other columns show the individual LAMP reactions and the detection channels of the thermocycler; FAM-FIP detected on green channel only and TAMRA-FIP by the orange channel.

With non-specific LAMP detection techniques that report on concentration changes of DNA or pyrophosphate production, there is limited potential for multiplexing. The specific detection of quenched fluorescence through labelling of FIP primers was investigated further to see if the technique can be multiplexed effectively. The FIP primer in the 35S promoter primer set was replaced with a TAMRA labelled FIP and ADH1 with FAM. Denatured maize genomic DNA with a transgenic proportion was assayed with each LAMP reaction individually and as a duplex. Displacement and Loop primers were not included to reduce non-specific primer interactions (Fig. 9).

In the duplex reaction ADH1 and 35Sp LAMP amplifications from the template are successfully detected by excitation and detection of the various fluorophores attached to the FIP primer in each set. The signal from FAM-FIP in the ADH1 LAMP amplification showed the least quenching of template amplification compared to the NTCs. The strongest signal was derived from the TAMRA-FIP used in the 35Sp LAMP amplification. Singleplex LAMP amplifications showed the correct identification in the green channel of FAM-FIP in the 35Sp LAMP amplification and appropriate negative results for the ADH1 LAMP amplifications. The orange channel selected the TAMRA labelled FIP in the 35Sp LAMP amplification and not the other LAMP amplifications.

## Discussion

We show here that FIP and LoopF labelled with JOE and FAM respectively can be successfully incorporated into LAMP reactions replacing their unlabelled counterparts and are quenched as a result of amplification. The mechanism by which the fluorescently labelled FIP and LoopF primers are quenched is unclear. One theory is that the formation of cauliflower-like concatamers in the elongation stage of LAMP amplification tightly packs the fluorophores to nucleotide residues in sufficiently close proximity for fluorescence resonance energy transfer (FRET) to quench the fluorescence as previously observed^35^. This phenomenon was observed with labelling of both the LoopF and FIP primers and with three different fluorophores; FAM, JOE and TAMRA. The FIP and BIP primers are the most fundamental to LAMP amplification because no other primers are required during LAMP cycling and elongation phases. We therefore used the FIP primers in our investigations, seeking to make use of the phenomenon.

The 10-fold dilutions of linearised plasmid template in Fig. 2 demonstrated the potential of the quenching primer method for high sensitivity and quantification by detecting 5 copies per microlitre in approximately 40 minutes and 5 million copies in approximately 20 minutes. However, at the lower concentration of DNA template the amplification frequencies were low for the two assays. Also variation of the quenched signals was evident for some of the template concentrations and the separation between non-template fluorescence and quenched fluorescence in the presence of template amplification was poor for some concentrations. Optimisation of the primer combinations and concentrations of the JOE labelled FIP in LAMP amplification was therefore carried out (Fig. 3), highlighting the optimal concentration from these experiments of 0.8 micromolar. At this concentration the variation between replicates was low and the difference between quenched and unquenched signals was clear.

We compared the quenching of the JOE-FIP primer to detection of LAMP amplification with SYTO9 including JOE-FIP in the primer set or with unlabelled FIP substituted (Fig. 4). All three methods showed high linearity to the semi logarithmic trendline, but it was observed that moderately faster times and lower variation were evident using the unlabelled FIP primer compared to the JOE-FIP assay. Nevertheless the observation that LAMP amplification with a fluorescently labelled FIP primer was successful with SYTO9 detection suggests that amplification could be monitored in real time on the green channel for SYTO9 and yellow channel for JOE of a qPCR thermocycler as a dual detection method.

Theoretically, the labelling of a FIP or BIP primer will have absolute specificity for the LAMP amplification. In the absence of template there would be no LAMP amplification and therefore no quenching of the signal from the fluorophore labelled LAMP primer. We sought to produce false positive results with a combination of a low purity LAMP primer set with extended reaction times. Detection of increases in double stranded DNA using the intercalating dye SYTO9 showed five positive partitions out of the 72 tested. The same assay monitored with JOE-FIP did not show quenching from any of the partitions. All false positives occurred after 150 minutes at 60 degrees C and are highly unlikely to be caused by contamination; positive template results were rapid and reaction times were similar for the two assays (Fig. 5).

By combining SYTO9 detection with JOE-FIP quenching in the same tube (Fig. 6) over an extended time period we could characterise the false positives generated in the absence of template by melt curve analysis and gel electrophoresis of the amplicons. Three false positives were evident in both the green channel for SYTO9 detection and the yellow channel for JOE-FIP quenching which indicated that the FIP primer was involved in the generation of the false positives. This was supported by a shift in melt temperature for these three and a ladder pattern on the agarose gel typical for LAMP amplification indicating concatamers of varying size. The closed tube format of this dual testing restricts amplicon contamination which is a risk when opening tubes for gel electrophoresis. The results also show the synchronicity of the false positives between the two methods reporting on a single partition.

We then explored the uses of quenching as a tool to optimise LAMP assays. Bioluminescent reporting of LAMP amplification with BART^25^ used to detect low concentrations of transgenes in genomic DNA samples shows a spread of positive results. Our investigation of the samples with quenching dual detection showed that the fastest groups of positive results are likely to be ‘true’ positives, but later results are likely to be false positives (Fig. 7). This was also supported with the melt curve analysis which showed a shift in melt temperatures between the ‘true’ positives and the false positives. This indicates that particular sets of LAMP primers are therefore susceptible to false positives at low copy number and would benefit from further investigation and possible redesign of certain primers. Displacement, LAMP and Loop primers can be designed using Primer Explorer (Eiken, Japan), commercial software and bioinformatic programmes^49,50^. However, the interactions between primers, target sequences, non-target sequences and amplicons are difficult to predict *in silico*. This demonstrates how quenched-based dual detection of amplification can serve as an optimisation tool in the development of LAMP assays.

Carrier DNA has previously been shown to enhance sensitivity, reduce variation between replicates, and increase reaction times and amplification frequency^9^ when added at 100 nanograms per reaction. We used the dual detection method with 35S promoter and NOS terminator LAMP amplification of a transgenic maize DNA sample to demonstrate that amplification frequency increased at 100 nanograms per reaction without an increase in false positive detection (Fig. 8). The fastest partition at each concentration of carrier DNA were of similar values whereas amplification frequency was highest at 100 nanograms reaction, possibly indicating that carrier DNA has a positive impact on initiating LAMP amplification from available target copies, but does not affect subsequent amplification.

Many fluorescent probe and modified primer technologies with enhanced specificity also describe adaptations for multiplexing. We have shown a duplex system here with JOE labelled LAMP primers and SYTO9, and we also investigated a duplex approach with the detection of reference gene ADH1 and transgene 35S promoter (Fig. 9). The FIP primers of each of these LAMP primer sets was linked to FAM and TAMRA respectively. Our results showed that two LAMP amplifications could occur in the same tube with quenching detection of the two labelled FIP primers. The excitation:emission for FAM is 495:520 and 544:576 for TAMRA. This duplex reaction shows that multiplexing with this technique could be developed.

We conclude that the simplicity of using quenching of a suitable 5′ labelled LAMP primer is an attractive proposition for specific LAMP detection and can be used with an intercalating dye to assess the extent to which false positives may affect a new LAMP primer assay. This will be an important factor in developing LAMP primers for digital LAMP assays due to the impact a false positive result could have on quantification calculations, and will enable existing indirect LAMP amplification detection methods to be employed with well designed, high quality primers.

## Methods

### DNA templates

A linearised plasmid pART7^51^, of defined copy number from calculations based on initial quantification by NanoDrop spectrophotometry and Agilent Bioanalyzer, was used as a DNA template. The plasmid contains a variant of the cauliflower mosaic virus 35S promoter sequence (35Sp). Genomic DNA was extracted from 0.1 percent w/w maize event Bt11 certified reference material (CRM) supplied by Fluka GmbH (Buchs, Switzerland). The transgenes in Bt11 are regulated by the 35S promoter from the cauliflower mosaic virus and the nopaline synthase terminator (NOSt) from *Agrobacterium tumefaciens*. The genomic maize DNA contains the alcohol dehydrogenase 1 gene sequence (ADH1). The Promega Wizard genomic DNA purification kit (Madison, United States) was used to extract the DNA from maize event Bt11 according to the manufacturer’s instructions for plant tissue. The final pellet was hydrated with 50 microlitres of rehydration buffer and stored at 4 degrees C. A further 5.0 percent Bt11 CRM, 5.0 percent NK603 CRM and 100 percent Mon810 maize sample were extracted using the Promega Wizard kit and quantified using the Qubit dsDNA BR assay kit (Thermofisher, Massachusetts USA) in accordance with the instructions for the Qubit 2.0 fluorometer.

### Copy number calculations

The initial quantification values from NanoDrop, Agilent Bioanalyzer and Qubit were converted from nanogram per microlitre to copies per microlitre using the following formula:

$${\rm{Copies}}\,{\rm{of}}\,{\rm{target}}=\frac{{\rm{ng}}\,{\rm{of}}\,{\rm{double}}\,{\rm{stranded}}\,{\rm{DNA}}\times {\rm{Avogadro}}\mbox{'}{\rm{s}}\,{\rm{constant}}\,({\rm{6.022}}\times {10}^{23})}{{\rm{Length}}\,{\rm{in}}\,{\rm{base}}\,{\rm{pairs}}\times {{\rm{10}}}^{{\rm{9}}}\times {\rm{650}}\,{\rm{Daltons}}}$$

The size of the linearised plasmid pART7 was assumed to be 4900 base pairs and 2.4 × 10(9) base pairs was used in calculations for the maize genome.

The online calculator was used at http://cels.uri.edu/gsc/cndna.html.

The calculations of copy number for the CRM materials for maize events Bt11 and NK603, and the seed stock of Mon810 are made in accordance with Hardinge *et al*.^46^ by adjusting transgenic copy numbers by 160/385. Futhermore Bt11 has two copies of the 35Sp promoter which doubles the available target sequence.

### Primer design and synthesis

Oligonucleotide primers for LAMP DNA amplification (Table 1) were synthesized and HPLC purified by Sigma Aldrich (Poole, UK). Primers were hydrated with molecular grade water to 100 micromolar and stored at minus 20 degrees C. The unmodified LAMP primers used to target 35Sp, NOSt and ADH1 sequences have previously been described and optimised^9,46,52^. An additional set of LAMP primers to target the 35S promoter was designed using Primer Explorer v4 from Eiken (Japan) for displacement, LAMP and loop primers. The stem primers were designed using the Primer 3 online software at http://primer3.ut.ee/. The fluorophores attached without additional modification to the 5′ end of FIP or LoopF primers were selected based on published evidence of nucleotide base quenching^35^. FAM, JOE and TAMRA were selected for LAMP quenching. Modifications to the 5′ terminal nucleotides were not made due to the NOSt JOE labelled FIP adjacent to a guanine base remained functional in amplification and LAMP quenching.

**Table 1:** Oligonucleotide primers for LAMP. Primers targetting the 35S promoter, NOS terminator and the ADH1 reference gene have been described previously and are attributed to Guy Kiddle (GK)^9^ and David Lee (DL)^52^.

Target, Type, Notation	Length	Primer Sequence (5′ to 3′)
35Sp, Displacement, F3	20	CTTATATAAGAGGAAGGGTCT
35Sp, Displacement, B3(P)	21	GATAAAGGAAAGGC**T**ATC**A**TT
35Sp, Displacement, B3(G)	20	ATAAAGGAAAGGCCATCGTT
35Sp, LAMP, FIP	39	CCACGTCTTCAAAGCAAGTGG-TTTT-GGATAGTGGGATTGTGCGTC
35Sp, LAMP, **JOE**-FIP	45	[**JOE**]CCACGTCTTCAAAGCAAGTGG-TTTT-GGATAGTGGGATTGTGCGTC
35Sp, LAMP, **TAMRA**-FIP	45	[**TAMRA**]CCACGTCTTCAAAGCAAGTGG-TTTT-GGATAGTGGGATTGTGCGTC
35Sp, LAMP, BIP	37	TTCCACGATGCTCCTCG-TTTT-CCTCTGCCGACAGTGG
35Sp, Loop, LoopF	16	TCCACTGACGTAAGGG
35Sp, Loop, **FAM**-LoopF	16	[**FAM**]TCCACTGACGTAAGGG
35Sp, Loop, LoopB	16	GGGGTCCATCTTTGGG
NOSt, Displacement, F3	22	CGCGATAATTTATCCTAGTTTG
NOSt, Displacement, B3	19	CGTTCAAACATTTGGCAAT
NOSt, LAMP, FIP	46	GCATGACGTTATTTATGAGA-TTTT-TCGCGCTATATTTTGTTTTCTA
NOSt, LAMP, **JOE**-FIP	46	[**JOE**]GCATGACGTTATTTATGAGA-TTTT-TCGCGCTATATTTTGTTTTCTA
NOSt, LAMP, BIP	43	CATGCTTAACGTAATTCAACA-TTTT-TGAATCCTGTTGCCGGTC
NOSt, Loop, LoopF	22	GATTAGAGTCCCGCAATTATAC
NOSt, Loop, LoopB	23	AAATTATATGATAATCATCGCAA
ADH1, Displacement, F3	19	CTTTGGATCGATTGGTTTC
ADH1, Displacement, B3	17	CCCAAAATTACTCAACG
ADH1, LAMP, FIP	48	CCCTCCGCAAATCTTCGAAC-TTTT-GTAACTGGTGAGGGACTGAG
ADH1, LAMP, **FAM**-FIP	48	[**FAM**]CCCTCCGCAAATCTTCGAAC-TTTT-GTAACTGGTGAGGGACTGAG
ADH1, LAMP, BIP	50	GGTGATCAAGTGCAAAGGTC-TTTT-CATAAACCAAGATTAGTCAGATCAAG
ADH1, Loop, LoopF	20	CGCCTTGTTTCTCCTCTGTC
ADH1, Loop, LoopB	16	CAATCCACTCCGAGAC

All primers are HPLC purified. The fluorophores used to label the FIP and LoopF primers were; 6-carboxyfluorescein (FAM), 6-carboxytetramethylrhodamine (TAMRA) and 6-carboxy-4′,5′-dichloro-2′,7′-dimethoxyfluorescein (JOE).

### Fluorescent LAMP amplification

DNA samples were amplified using LAMP and detected using SYTO9 in real time on a Qiagen (Hilden, Germany) RotorGene thermal cycler acquiring to the green channel unless otherwise specified. All reagents were supplied by Sigma Aldrich (Poole, UK) unless otherwise stated. The reaction chemistry for LAMP and amplification detection was 1X isothermal buffer (New England Biolabs Inc, Massachusetts, United States), 300 micromolar each deoxynucleotide triphosphate (dNTP), 0.8 micromolar Betaine, 0.32 units per microlitre Bst polymerase v2.0 warm start (NEB), 0.5 micromolar SYTO9 Green, 0.8 micromolar each LAMP primer, 0.4 micromolar each Loop primer, 0.2 micromolar each displacement primer and molecular grade water for a reaction volume of 20 microlitres. The parameters were set for 60 cycles of 60 seconds at 60 degrees C unless otherwise stated. Temperature melt analysis provided data between 60 and 95 degrees C. Results were analysed on RotorGene 6000 software v1.7 and Microsoft Excel. The threshold was set at the mid point between the background fluorescence and quenched fluorescence from the positive samples. The default threshold value was −0.05 on the normalised fluorescence scale. Standard normalisation was used in the RotorGene software to take the average background from the first five cycles to provide a value by which sample data points are divided.

### Quenched fluorescence LAMP amplification

Oligonucleotide LAMP primers with JOE, FAM or TAMRA fluorophores attached to the 5 prime end were incorporated into the fluorescent LAMP amplification method without the inclusion of SYTO9. For dual detection SYTO9 was included with JOE labelled FIP primers. The LAMP reaction was monitored in real time on the RotorGene thermocycler according the emission wavelength of the fluorophore. The multiplex and dual detection reactions monitored 6′FAM or SYTO9 using the green channel (source 470 +/− 10 nanometers, detection 510 +/− 5 nanometers), JOE on the yellow channel (source 530 +/− 5 nanometers, detection 557 +/− 5 nanometers) and TAMRA with the orange channel (excitation at 585 +/− 5 nanometers, detection aat 610 +/− 5 nanometers) in the same reaction. Melt temperature analysis followed amplification between 60 and 95 degrees C. Results were analysed RotorGene 6000 software v1.7 and Microsoft Excel.

### Real-time LAMP-BART detection

LAMP-BART chemistry was previously described by Gandelman *et al*.^25^ and Kiddle *et al*.^9^. Salmon sperm carrier DNA at 100 nanograms per partition was used in partitions of 5 microlitres. The LAMP-BART reaction mix contained 1x Thermopol buffer (NEB), 10 millimolar dithiothrietol (DTT), 0.4 milligrams per millilitre polyvinylpyrrolidone (PVP), 60 millimolar potassium chloride (KCl), 300 micromolar each dNTP, 100 micrograms per millilitre D-luciferin (Europa, Ipswich, UK), 250 micromolar adenosine-5′-O-phosphosulfate (APS; Biolog, Bremen, Germany), 5.5 micrograms per millilitre Ultra-Glo luciferase (Promega, Madison, United States), 0.375 units per microlitre ATP sulfurylase (NEB), 0.32 units per microlitre Bst polymerase v1.0, 0.8 micromolar each LAMP primer, 0.4 micromolar each Loop primer, 0.2 micromolar each displacement primer and molecular grade water. Each partition was layered with mineral oil and sealed with a clear adhesive film. The bioluminescent signal was detected and analysed using React IVD software (Synoptics, Cambridge, UK) in a ‘Lucy’ device developed by ERBA MDX (Ely, UK). The LAMP-BART assays were set for 90 minutes at 60 degrees C.

## Supplementary information

Supplementary Information

## Data Availability

The datasets generated during and/or analysed during the current study are available from the corresponding author on reasonable request.
