# Supplementary material for: Reduced False Positives and Improved Reporting of Loop-Mediated Isothermal Amplification using Quenched Fluorescent Primers
Source: Sci Rep. 2019 May 14;9:7400. doi: 10.1038/s41598-019-43817-z (PMC6517417; doi:10.1038/s41598-019-43817-z)
Supplement: Supplementary file 1 — Supplementary Information [file 41598_2019_43817_MOESM1_ESM.docx]

Supplementary Information

**Reduced False Positives and Improved Reporting of Loop-Mediated Isothermal Amplification using Quenched Fluorescent Primers**

**Patrick Hardinge^1,*^, James A. H. Murray^1^**

^1^Cardiff School of Biosciences, Biomedical Sciences Building, Museum Avenue, Cardiff CF10 3AX, UK

*corresponding author: hardingep@cardiff.ac.uk


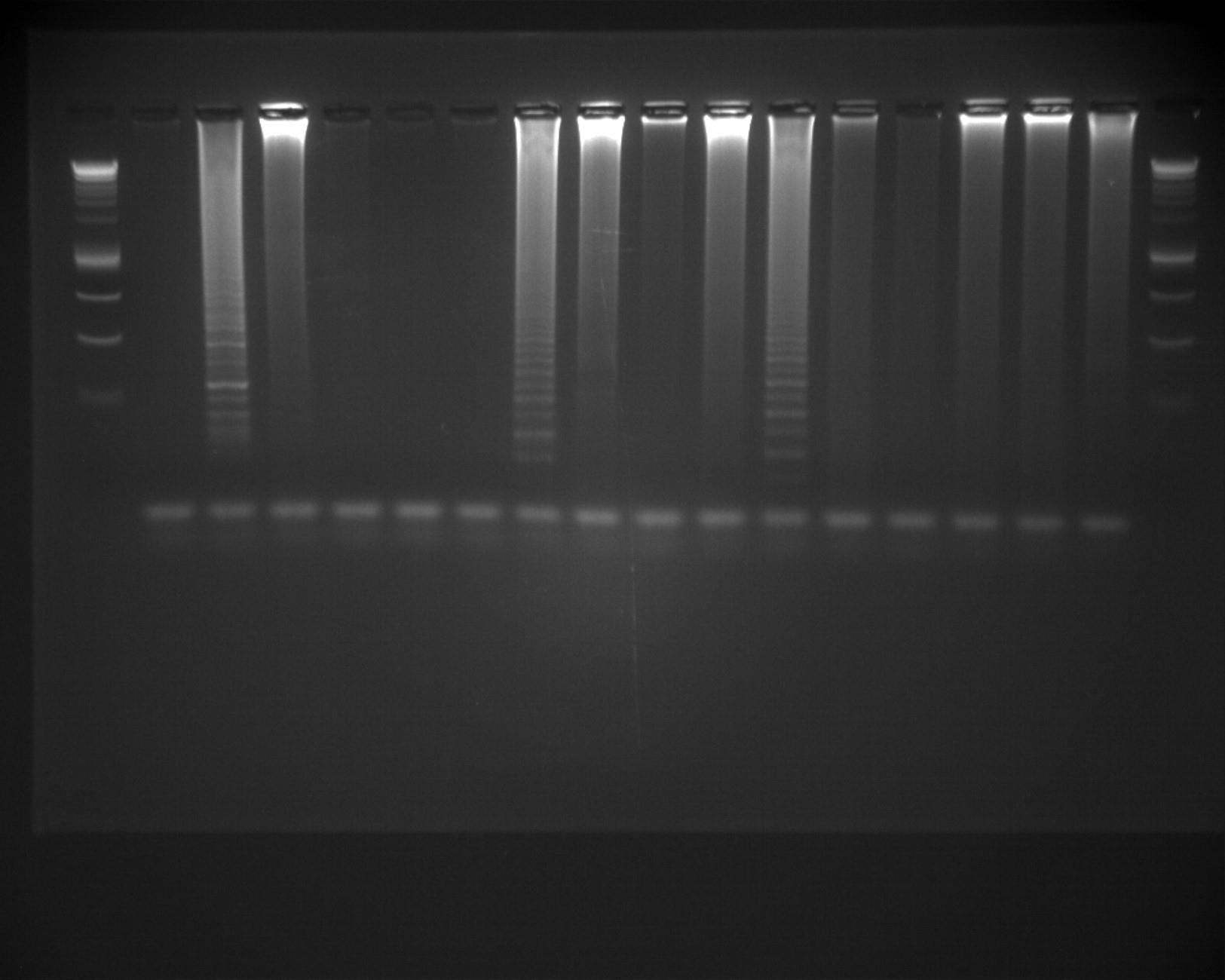


**Figure S1. Full length agarose gel used in Figure 6.**


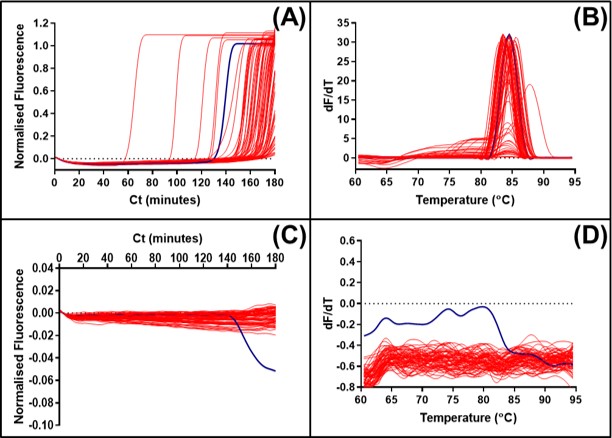


**Figure S2. Non-specific primer interactions detected with SYTO9 and JOE-FIP.** Panel (A) shows the normalised fluorescence from SYTO9 detection and (C) shows the consecutive JOE-FIP fluorescence for non-template controls with new 35Sp LAMP primers. Corresponding melt curve analysis shown in panels (B) and (D). Negative results and SYTO9 detected positives are highlighted in red, JOE-FIP detected positives are highlighted in blue.

Basic purified Sigma synthesised LAMP oligonucleotides targeting the 35Sp sequence and designed using Primer Explorer v4 were used in combination to increase the incidence of false positive results.

BIP: 5’ CACGTCTTCAAAGCAAGTGGTTTTATAGTGGGATTGTGCGTCATC 3’, F3: 5’ TGGCTCCTAGAAATGCCATC 3’, B3: 5’ GAGGAAGGGTCTTGCGAAG 3’, LoopB: 5’ TGATGTGATATCTCCACTGAC 3’, StemB: 5’ CGATGCTCCTCGTGGGTGGG 3’. The JOE-FIP primer 5’ ATCTTTGGGACCACTGTCGTTTTATTGCGATAAAGGAAAGGC 3’ was HPLC purified.


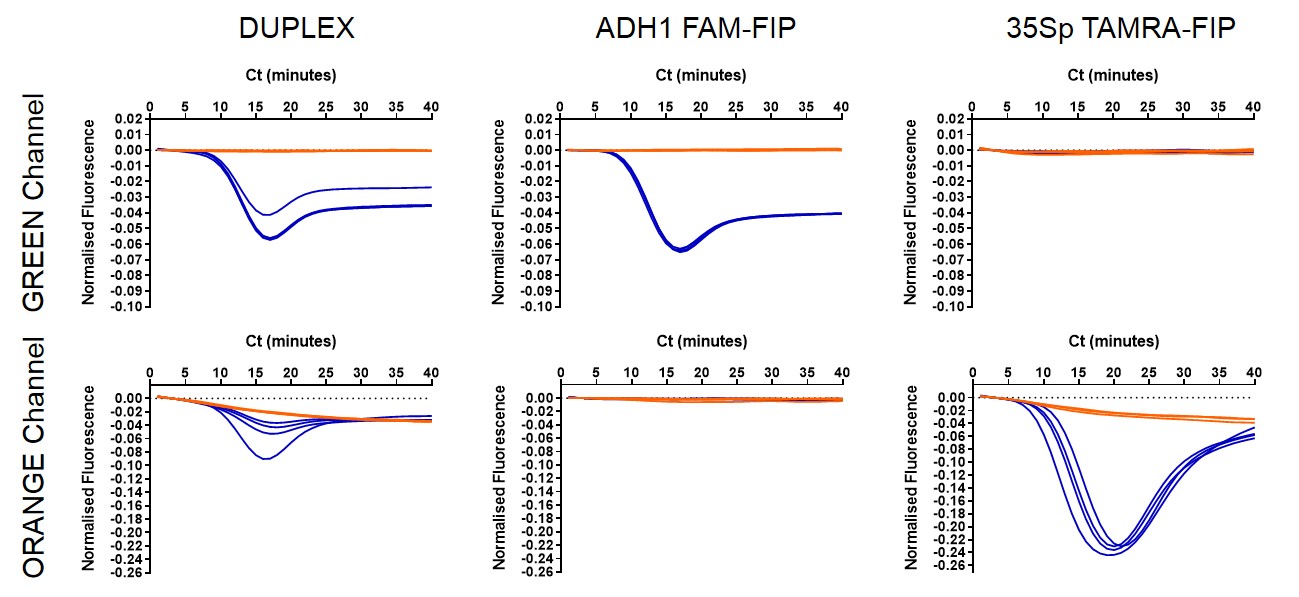


**Figure S3. Duplex LAMP reaction with ADH1 and 35Sp detection.** LAMP amplification and detection of 1in10 dilution of native **5% Bt11** maize genomic DNA with ADH1 LAMP primers with FAM labelled FIP and 35Sp LAMP primers with TAMRA labelled FIP. Qubit quantification and copy number calculations: 1096 copies per microlitre ADH1, 46 copies per microlitre 35Sp. Blue indicates template amplification and orange denotes the NTCs. The (DUPLEX) column shows the detection of two LAMP reactions in each tube. Other columns show the individual LAMP reactions and the detection channels of the thermocycler; FAM-FIP detected on the green channel only and TAMRA-FIP by the orange channel only.


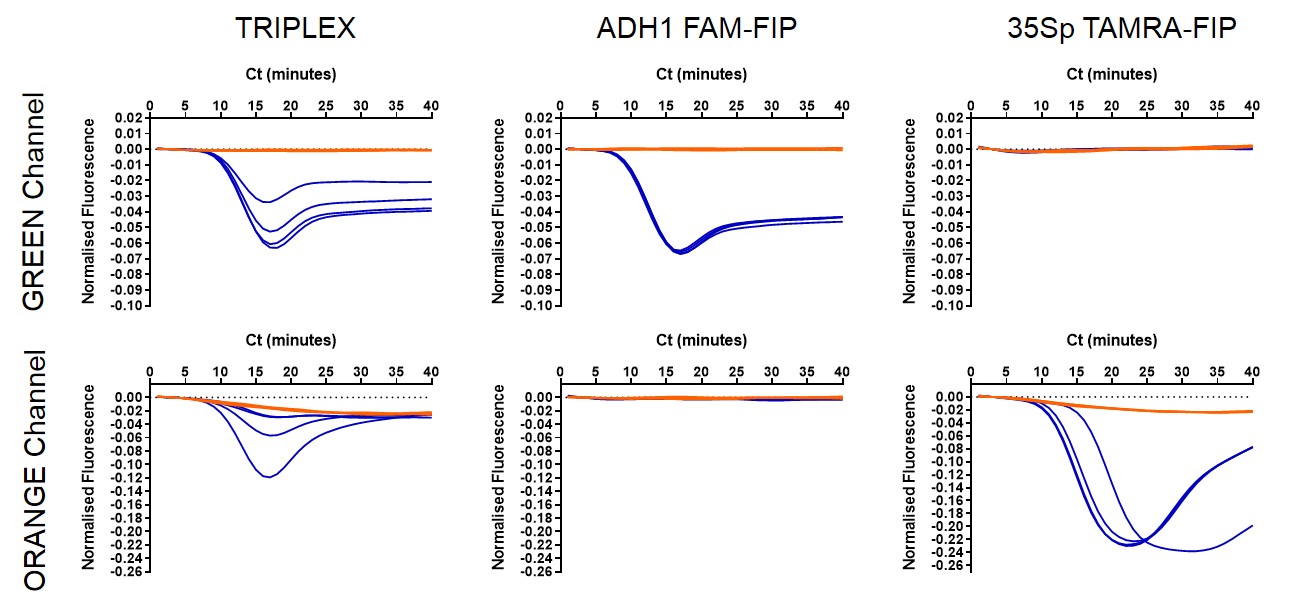


**Figure S4. Duplex LAMP reaction with ADH1 and 35Sp detection.** LAMP amplification and detection of 1in10 dilution of native **5% NK603** maize genomic DNA with ADH1 LAMP primers with FAM labelled FIP and 35Sp LAMP primers with TAMRA labelled FIP. Qubit quantification and copy number calculations: 1073 copies per microlitre ADH1, 22 copies per microlitre 35Sp. Blue indicates template amplification and orange denotes the NTCs. The (DUPLEX) column shows the detection of two LAMP reactions in each tube. Other columns show the individual LAMP reactions and the detection channels of the thermocycler; FAM-FIP detected on the green channel only and TAMRA-FIP by the orange channel only.


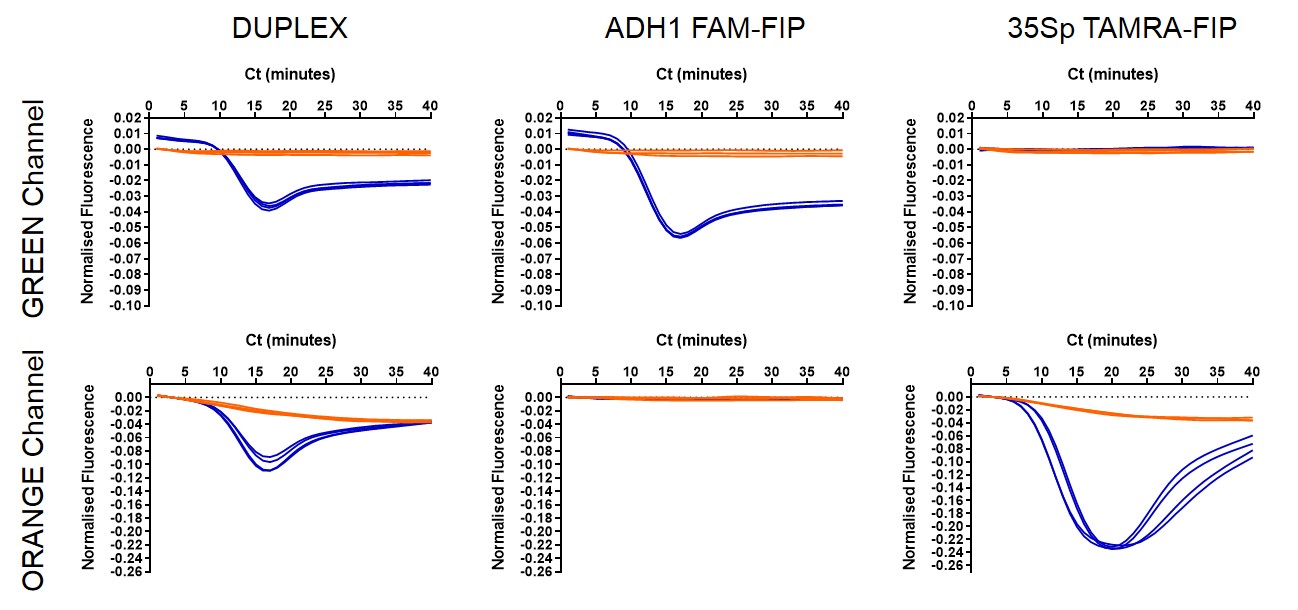


**Figure S5. Duplex LAMP reaction with ADH1 and 35Sp detection.** LAMP amplification and detection of 1in10 dilution of native **100% Mon810** maize genomic DNA with ADH1 LAMP primers with FAM labelled FIP and 35Sp LAMP primers with TAMRA labelled FIP. Qubit quantification and copy number calculations: 1745 copies per microlitre ADH1, 725 copies per microlitre 35Sp. Blue indicates template amplification and orange denotes the NTCs. The (DUPLEX) column shows the detection of two LAMP reactions in each tube. Other columns show the individual LAMP reactions and the detection channels of the thermocycler; FAM-FIP detected on the green channel only and TAMRA-FIP by the orange channel only.


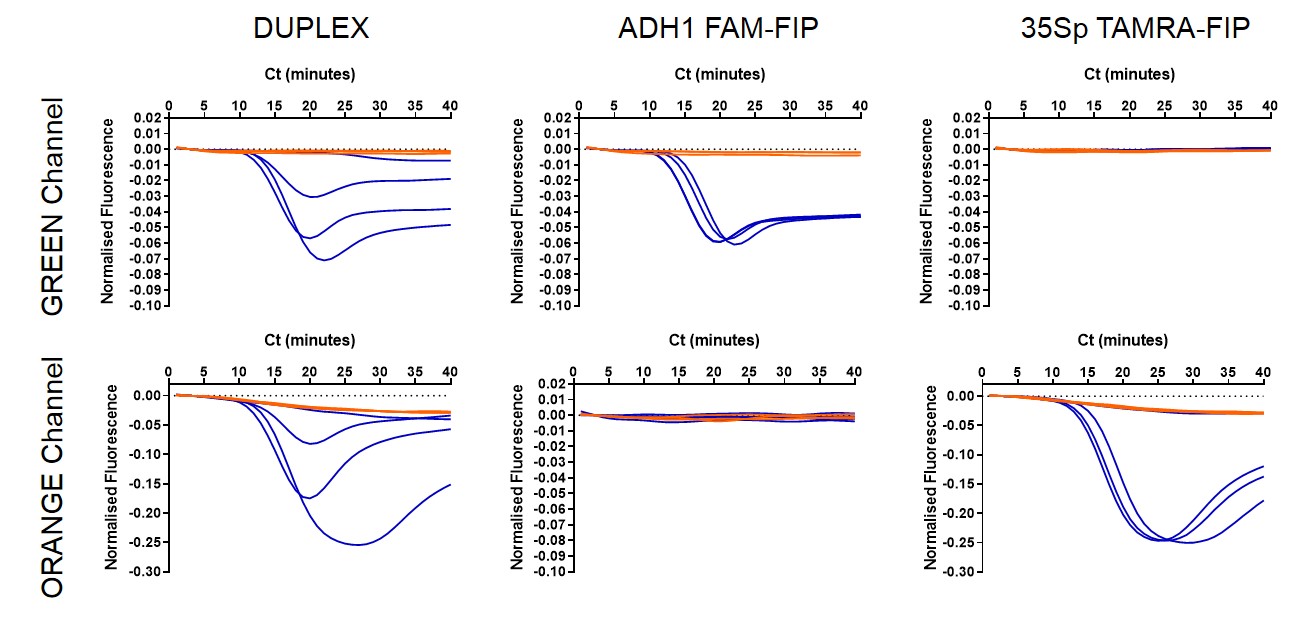


**Figure S6. Duplex LAMP reaction with ADH1 and 35Sp detection.** LAMP amplification and detection of 1in1000 dilution of native **100% Mon810** maize genomic DNA with ADH1 LAMP primers with FAM labelled FIP and 35Sp LAMP primers with TAMRA labelled FIP. Qubit quantification and copy number calculations: 17 copies per microlitre ADH1, 7 copies per microlitre 35Sp. Blue indicates template amplification and orange denotes the NTCs. The (DUPLEX) column shows the detection of two LAMP reactions in each tube. Other columns show the individual LAMP reactions and the detection channels of the thermocycler; FAM-FIP detected on the green channel only and TAMRA-FIP by the orange channel only.
